# An oral microbiome model for predicting atherosclerotic cardiovascular disease

**DOI:** 10.3389/fcimb.2025.1707599

**Published:** 2026-01-26

**Authors:** Qiuli Sui, Jie Yu, Shuping Cui

**Affiliations:** 1 Department of Stomatology, People's Hospital of Zhengzhou University & Henan Provincial People's Hospital, Zhengzhou, China; 2 Department of Pediatric Dentistry, Hangzhou Stomatology Hospital Pinghai Branch, Hangzhou, China

**Keywords:** atherosclerosis, cardiovascular disease prevention, machine learning, oral microbiome, predictive model

## Abstract

**Objective:**

This study aimed to construct a predictive model for the early onset of atherosclerotic cardiovascular disease (ASCVD) by integrating oral microbiome data with traditional clinical risk factors.

**Methods:**

A retrospective study was conducted involving participants aged 50–70 years without pre-existing ASCVD. The patients were divided into a training set and a validation set at a ratio of 7:3 by the complete randomization method. The characteristics of the oral microbiome were characterized by 16S rRNA/metagenomic sequencing. In the training set, univariate analysis and multivariate Logistic regression analysis were applied to screen predictive variables, and Random Forest (RF), Gradient Boosting (GB), and K-nearest Neighbor (KNN) were constructed. The receiver operating characteristic (ROC) curve was validated. The model performance was evaluated by net reclassification improvement (NRI) and integrated discrimination improvement (IDI).

**Results:**

A total of 331 patients were enrolled and randomly divided into a training set (n=231) and a validation set (n=100). 40 out of 331 participants experienced major adverse cardiovascular events (MACE). Multivariate Logistic regression analysis confirmed that age, relative abundance of *Fusobacterium nucleatum, Prevotella, Porphyromonas*, *Leptotrichia*, *Streptococcus* and *Actinomyces* were significantly associated with ASCVD event risk (all *P* < 0.05). Three machine learning models (RF, GB, and KNN) were constructed, with the RF model achieving the highest predictive performance. The AUC values of the RF, GB, and KNN models in the training set were 0.888 (95% CI: 0.818-0.958), 0.823 (95% CI: 0.745-0.901), and 0.812 (95% CI: 0.727-0.898) respectively, and in the validation set were 0.845 (95% CI: 0.740-0.951), 0.746 (95% CI: 0.621-0.871), and 0.767 (95% CI: 0.647-0.887) respectively. Additionally, the integrated model showed significant improvements in net reclassification improvement (NRI = 0.315, P < 0.05) and integrated discrimination improvement (IDI = 0.227, P < 0.05) compared to traditional clinical models.

**Conclusion:**

The integration of the oral microbiome and clinical data can improve the accuracy of the ASCVD risk prediction model, providing a novel biomarker strategy for primary cardiovascular prevention.

## Introduction

Atherosclerotic Cardiovascular Disease (ASCVD) was one of the leading causes of death and disability worldwide, resulting in over 17 million deaths annually and imposing a heavy burden on public health systems (Zhang et al., 2022). Currently, traditional risk assessment models widely used in clinical practice, such as the Framingham Risk Score and Pooled Cohort Equations, primarily rely on indicators including age, lipid levels (e.g., low-density lipoprotein cholesterol, LDL-C), blood pressure, smoking status, and diabetes history (Rout et al., 2024). However, these models have obvious limitations: on one hand, approximately 20%-30% of individuals classified as “intermediate risk” by traditional models still experience ASCVD events, indicating insufficient accuracy in risk stratification (Mousavi et al., 2025). On the other hand, they do not incorporate non-traditional biomarkers, making it difficult to capture early pathophysiological changes of the disease. Therefore, there was an urgent need to explore new and reliable predictive indicators to optimize ASCVD primary prevention strategies.

As one of the most microbially rich sites in the human body (second only to the intestine), the oral cavity and the imbalance of its microbial homeostasis have become a research hotspot in recent years due to their association with systemic diseases (Barbour et al., 2022). Existing studies have confirmed that the oral microbiota can participate in disease development through the “oral-systemic” axis: cross-sectional studies have detected DNA of oral pathogenic bacteria such as Porphyromonas gingivalis and Fusobacterium nucleatum in atherosclerotic plaques, and these bacteria can remain at the site of vascular endothelial damage through the bloodstream, directly promoting lipid deposition and plaque formation (Nearing et al., 2020; Linton et al., 2023). Nibali et al. further found that changes in the abundance of genera such as Streptococcus and Actinomyces were significantly correlated with metabolic syndrome (a key risk factor for ASCVD), suggesting that the oral microbiota may indirectly affect cardiovascular health by regulating systemic inflammatory responses (e.g., increasing IL-6 and TNF-α levels) (Nibali et al., 2023). In addition, Nibali et al. found through echocardiography that periodontal probing depth (reflecting the degree of oral inflammation) was positively correlated with left ventricular hypertrophy (a manifestation of ASCVD target organ damage), further verifying the “oral inflammation-cardiovascular damage” association pathway (Nibali et al., 2019). However, current evidence has obvious shortcomings: most studies adopt a cross-sectional design, which can only confirm “association” but not “predictability”; a small number of studies involving predictive value have small sample sizes (<200 cases) and do not combine efficient variable screening methods such as machine learning, resulting in the unclear clinical value of oral microbiota as an ASCVD predictive biomarker and forming a critical research gap (Nearing et al., 2020).

Based on the above research status and shortcomings, this study proposes a core hypothesis: baseline oral microbiome characteristics (including microbial αicrobialgs and relative abundance of specific genera) are independent predictors of future ASCVD events, and integrating these microbial markers with traditional clinical risk factors can significantly improve the accuracy of ASCVD risk prediction models. The specific research objectives of this study include: (1) clarifying the association strength between baseline oral microbiota and the occurrence of ASCVD events during follow-up through a retrospective cohort study design; (2) screening oral microbial markers with robust predictive value using machine learning algorithms such as Least Absolute Shrinkage and Selection Operator (LASSO) regression combined with Random Forest (RF) and Gradient Boosting (GB); (3) constructing an integrated prediction model of “traditional clinical indicators + oral microbiota” and verifying its predictive performance through Net Reclassification Improvement (NRI) and Integrated Discrimination Improvement (IDI), so as to provide a new tool for the early identification of high-risk populations with ASCVD.

## Materials and methods

### Study population

This study was a retrospective study. From January 2019 to December 2020, a total of 331 community subjects were recruited. Inclusion criteria: no clinically diagnosed ASCVD (including myocardial infarction, stroke, revascularization, etc.) at baseline (Barbour et al., 2022); aged 50–70 years; having complete baseline data of oral microbiome, clinical biochemical indicators, and clinical evaluations; consenting to participate in long-term follow-up and providing valid contact information. Exclusion criteria: a history of ASCVD; suffering from severe systemic diseases; use of systemic antibiotics within the past 3 months; periodontal treatment within the past 1 month; fewer than 10 functional teeth; being pregnant or lactating; or anticipated difficulty in completing follow-up.

### Data collection

Demographic data, lifestyle, and disease history were collected through standardized questionnaires. Blood pressure, height, and weight were measured by trained medical staff, and the body mass index (BMI) was calculated. Fasting venous blood was collected from all participants for the detection of total cholesterol (TC), low-density lipoprotein cholesterol (LDL-C), high-density lipoprotein cholesterol (HDL-C), triglycerides (TG), fasting plasma glucose (FPG), high-sensitivity C-reactive protein (hs-CRP), and other indicators. Unstimulated saliva samples (2 mL) were collected from all participants using a sterile saliva collector and stored at-80 °C until DNA extraction (Hoogeveen and Ballantyne, 2021). The V3-V4 hypervariable region of the 16S rRNA gene was PCR-amplified and sequenced on the Illumina MiSeq platform (Choi and Hong, 2026). The original sequencing data were quality-controlled, denoised, spliced, and chimeras were removed using the QIIME 2 and DADA2 pipelines, and finally, an Amplicon Sequence Variants (ASVs) table was obtained. Species annotation was performed based on ASVs, and α-diversity (such as the Shannon index) and β-diversity (PCoA analysis based on Bray-Curtis distance) were calculated (Kaan et al., 2021). Note: The relative abundance data of oral microbiota were used directly in subsequent statistical and machine learning models without additional transformation (e.g., centered log-ratio transformation), and specific parameters such as rarefaction depth and average reads per sample will be supplemented in future data collation.

### Outcome definition

According to internationally recognized clinical research standards, the primary endpoint event was defined as the first occurrence of major adverse cardiovascular events (MACE) (Ogbanga et al., 2023), and the subjects were grouped accordingly. The event group was defined as patients who experienced MACE (including non-fatal myocardial infarction, non-fatal myocardial infarction, coronary revascularization, and cardiovascular-related death) during the follow-up period; the non-event group consisted of control subjects who did not experience the above events during the follow-up.

### Sample size estimation

Sample size estimation followed the events-per-variable (EPV) principle, with a target EPV ≥ 5. It was estimated that based on preliminary data, previous literature reviews, and clinical experience, the estimated incidence of MACE among respondents with primary cardiovascular endpoint events during the follow-up period was approximately 20% (Kunath et al., 2024). In the final multiple logistic regression model, about 7–10 predictive variables were planned to be included. Therefore, the minimum number of required events according to the principle of EPV≥5: E (required number of events) =EPV×V =10 × 5 = 50 events. Based on the estimated event incidence (P ≈ 20%), the total sample size required was estimated to be at least: 50 (events)/0.20 (incidence)≈250 patients. Considering that there might be about 20% data loss or exclusion during the study (such as poor serum sample quality and incomplete clinical data), the sample size was increased by 20% on this basis, and the final target sample size was: 250/(1-0.2)≈312 patients. The three-year patient cohort (from January 2019 to December 2020) included in this study exceeded this limit. The target sample size was set to ensure sufficient statistical validity of this study.

### Statistical analysis

Statistical analysis was performed using SPSS 26.0, Python 3.8.5 and R 4.2.3. Measurement data conforming to the normal distribution were expressed as x̄ ± s, and the t-test was used for comparison between groups. Data not conforming to the normal distribution were expressed as the median (interquartile range), and the Mann-Whitney U test was used. Count data were expressed as the number of cases (percentage), and the *χ²* test was used for comparison between groups. In the training set, univariate analysis was first performed to screen out indicators with *P* < 0.05. After variable compression by LASSO regression, multivariate logistic regression analysis was used to determine independent influencing factors. Before model construction, continuous variables (including clinical indicators and microbial relative abundance) were standardized using z-score normalization to eliminate the impact of dimensional differences. Random forest (RF), Gradient Boosting (GB), and K-nearest neighbor (KNN) algorithm models were constructed based on Python 3.8.5 (using scikit-learn package). For parameter optimization: 1) RF model: The number of trees was set to 500, and mtry was optimized to 3 through grid search (tested values: 1-10); 2) GB model: Learning rate was optimized to 0.1, tree depth to 3, and number of trees to 300 via grid search; 3) KNN model: k value was optimized to 5 using cross-validation (tested values: 3-15). During model training, 5-fold cross-validation was used, and nested cross-validation was employed to verify the model performance to avoid over-optimistic estimates. The receiver operating characteristic (ROC) curve was plotted, and the area under the curve (AUC) value was calculated. The model performance was evaluated by net reclassification improvement (NRI) and integrated discrimination improvement (IDI). A P value < 0.05 was considered statistically significant.

## Results

### Comparison of baseline characteristics in the training and validation sets

**Figure 1:**
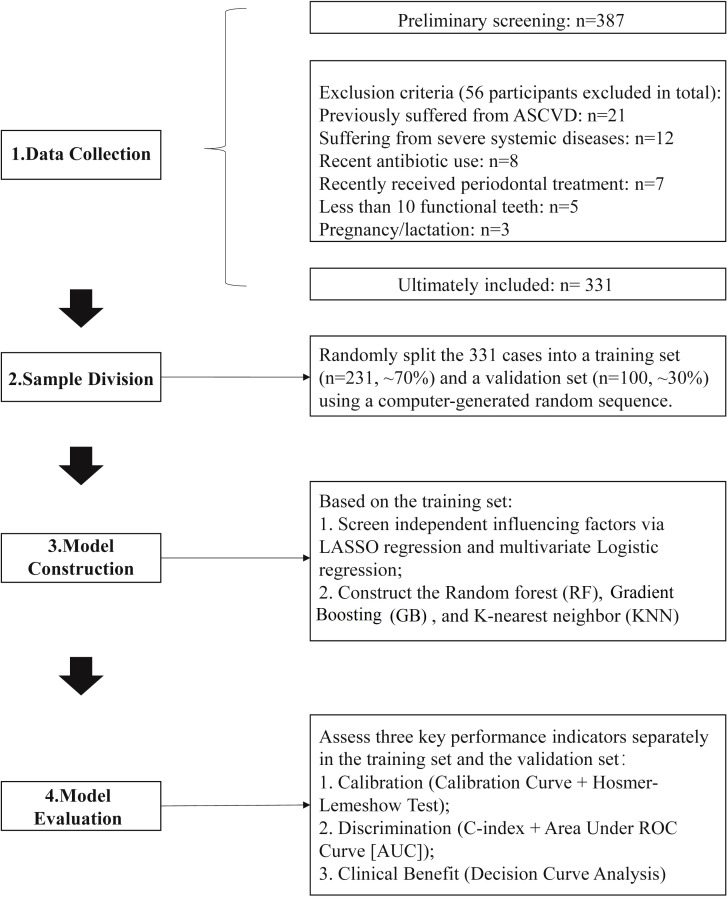
Recruitment flow diagram of the study.

A total of 387 patients were initially screened from January 2019 to December 2020, 56 subjects were excluded. Finally, 331 patients were enrolled and randomly divided into a training set (n=231) and a validation set (n=100) (**Figure 1**). The training set included 231 patients, with 184 cases (79.65%) in the non-event group and 47 cases (20.35%) in the event group. The validation set comprised 100 patients, of whom 80 cases (80.00%) in the non-event group and 20 cases (20.00%) in the event group. There were no statistically significant differences in the baseline characteristics of patients between the training set and the validation set (*P* > 0.05) (**Table 1**).

**Table 1:** Comparison of baseline characteristics in the training and validation sets.

Indicators	Training set (n = 231)	Validation set (n = 100)	*t/χ²*	*P*
Age (years)	63.71 ± 5.84	63.52 ± 9.13	0.227	0.821
Gender (Male/Female)	135(58.44)/96(41.56)	56(56.00)/44(44.00)	0.171	0.680
BMI (kg/m²)	26.31 ± 4.20	26.87 ± 3.95	1.134	0.256
Systolic blood pressure	132.51 ± 16.33	134.17 ± 15.79	0.858	0.392
Diastolic blood pressure	82.43 ± 10.51	81.97 ± 9.99	0.371	0.711
Total cholesterol	5.12 ± 1.05	5.04 ± 0.93	0.658	0.511
Low-density lipoprotein cholesterol	3.18 ± 0.89	3.25 ± 0.85	0.666	0.506
High-density lipoprotein cholesterol	1.25 ± 0.31	1.22 ± 0.33	0.793	0.429
Triglycerides	1.68 ± 0.92	1.75 ± 0.87	0.646	0.519
Fasting blood glucose	5.90 ± 1.63	5.81 ± 1.54	0.469	0.640
Diabetes (Yes/No)	63(27.27)/168(72.73)	23(23.00)/77(77.00)	0.663	0.416
History of hypertension (Yes/No)	110(47.62)/121(52.38)	43(43.00)/57(57.00)	0.599	0.439
Current smoking status (Yes/No)	77(33.33)/154(66.67)	30(30.00)/70(70.00)	0.355	0.552
Dyslipidemia (Yes/No)	98(42.42)/133(57.58)	41(41.00)/59(59.00)	0.058	0.810
Obesity (Yes/No)	65(28.14)/166(71.86)	29(29.00)/71(71.00)	0.026	0.873
Metabolic syndrome (Yes/No)	52(22.51)/179(77.49)	21(21.00)/79(79.00)	0.093	0.701
High-sensitivity C-reactive protein	2.35 ± 1.82	2.51 ± 1.90	0.725	0.469
Neutrophil-lymphocyte ratio	2.21 ± 0.87	2.18 ± 0.91	0.284	0.777
Platelet-lymphocyte ratio	149.83 ± 61.54	142.11 ± 56.79	1.072	0.284
Estimated glomerular filtration rate	87.24 ± 16.83	89.35 ± 15.82	1.066	0.287
Serum creatinine	79.51 ± 19.30	77.69 ± 18.06	0.803	0.423
Serum albumin	38.52 ± 5.03	39.21 ± 4.59	1.176	0.241
Shannon diversity index	4.15 ± 0.63	4.09 ± 0.60	0.807	0.420
Relative abundance of Fusobacterium nucleatum	1.58 ± 1.22	1.48 ± 1.15	0.697	0.487
Relative abundance of Prevotella	0.95 ± 0.71	0.89 ± 0.67	0.718	0.473
Relative abundance of Porphyromonas	0.45 ± 0.38	0.41 ± 0.35	0.900	0.369
Relative abundance of Leptotrichia	0.62 ± 0.55	0.58 ± 0.22	0.703	0.483
Relative abundance of Streptococcus	8.85 ± 4.65	9.20 ± 4.82	0.622	0.535
Relative abundance of Rothia	2.10 ± 1.58	2.25 ± 1.62	0.787	0.432
Relative abundance of Actinomyces	1.83 ± 1.40	1.91 ± 1.45	0.472	0.637

### Univariate analysis of factors associated with cardiovascular endpoint events

Univariate analysis showed that in the training set, there were statistically significant differences in age, LDL-C, current smoking status, relative abundance of *Fusobacterium nucleatum*, *Prevotella*, *Porphyromonas*, *Leptotrichia*, *Streptococcus*, *Rothia*, and *Actinomyces* between the non-event group and the event group (*P* < 0.05) (**Table 2**).

**Table 2:** Univariate analysis of influencing factors in the training set.

Indicators	Non-event group (n = 184)	Event group (n = 47)	*t/χ²*	*P*
Age (years)	62.81 ± 5.54	65.79 ± 5.87	3.251	0.001
Gender (Male/Female)	105(57.07)/79(42.93)	31(65.96)/16(34.04)	1.223	0.269
BMI (kg/m²)	26.41 ± 4.20	26.33 ± 3.89	0.118	0.906
Systolic blood pressure	131.84 ± 16.53	136.79 ± 14.81	1.870	0.063
Diastolic blood pressure	82.14 ± 10.81	83.79 ± 9.56	0.955	0.341
Total cholesterol	5.08 ± 1.08	5.32 ± 0.88	1.408	0.161
Low-density lipoprotein cholesterol	3.05 ± 0.82	3.50 ± 0.91	3.282	0.001
High-density lipoprotein cholesterol	1.26 ± 0.32	1.20 ± 0.28	1.175	0.241
Triglycerides	1.65 ± 0.95	1.88 ± 0.78	1.532	0.127
Fasting blood glucose	5.81 ± 1.53	6.24 ± 1.86	1.643	0.102
Diabetes (Yes/No)	48(26.09)/136(73.91)	15(31.91)/32(68.09)	0.641	0.423
History of hypertension (Yes/No)	85(46.20)/99(53.80)	26(55.32)/21(44.68)	1.248	0.264
Current smoking status (Yes/No)	53(28.80)/131(71.20)	25(53.19)/22(46.81)	9.956	0.002
Dyslipidemia (Yes/No)	72 (39.13)/112 (60.87)	25 (53.19)/22 (46.81)	3.039	0.081
Obesity (Yes/No)	50 (27.17)/134 (72.83)	15 (31.91)/32 (68.09)	0.416	0.519
Metabolic syndrome (Yes/No)	38 (20.65)/146 (79.35)	14 (29.79)/33 (70.21)	1.791	0.181
High-sensitivity C-reactive protein	2.28 ± 1.75	2.82 ± 2.12	1.805	0.072
Neutrophil-lymphocyte ratio	2.18 ± 0.84	2.38 ± 1.05	1.381	0.169
Platelet-lymphocyte ratio	148.23 ± 60.19	157.52 ± 67.34	0.921	0.358
Estimated glomerular filtration rate	85.23 ± 14.96	83.94 ± 15.78	0.522	0.602
Serum creatinine	78.26 ± 18.45	79.92 ± 17.51	0.556	0.579
Serum albumin	38.64 ± 5.19	38.06 ± 4.59	0.699	0.485
Shannon diversity index	4.12 ± 0.65	4.28 ± 0.51	1.568	0.118
Relative abundance of Fusobacterium nucleatum	1.35 ± 1.05	1.94 ± 1.25	3.303	0.001
Relative abundance of Prevotella	0.82 ± 0.60	1.16 ± 0.75	3.287	0.001
Relative abundance of Porphyromonas	0.38 ± 0.32	0.56 ± 0.40	3.262	0.001
Relative abundance of Leptotrichia	0.55 ± 0.45	0.82 ± 0.65	3.326	0.001
Relative abundance of Streptococcus	9.45 ± 4.51	7.11 ± 4.13	3.227	0.001
Relative abundance of Rothia	2.25 ± 1.52	1.45 ± 1.23	3.338	0.001
Relative abundance of Actinomyces	1.95 ± 1.35	1.25 ± 1.05	3.307	0.001

### Multivariate logistic regression analysis of influencing factors

**Figure 2:**
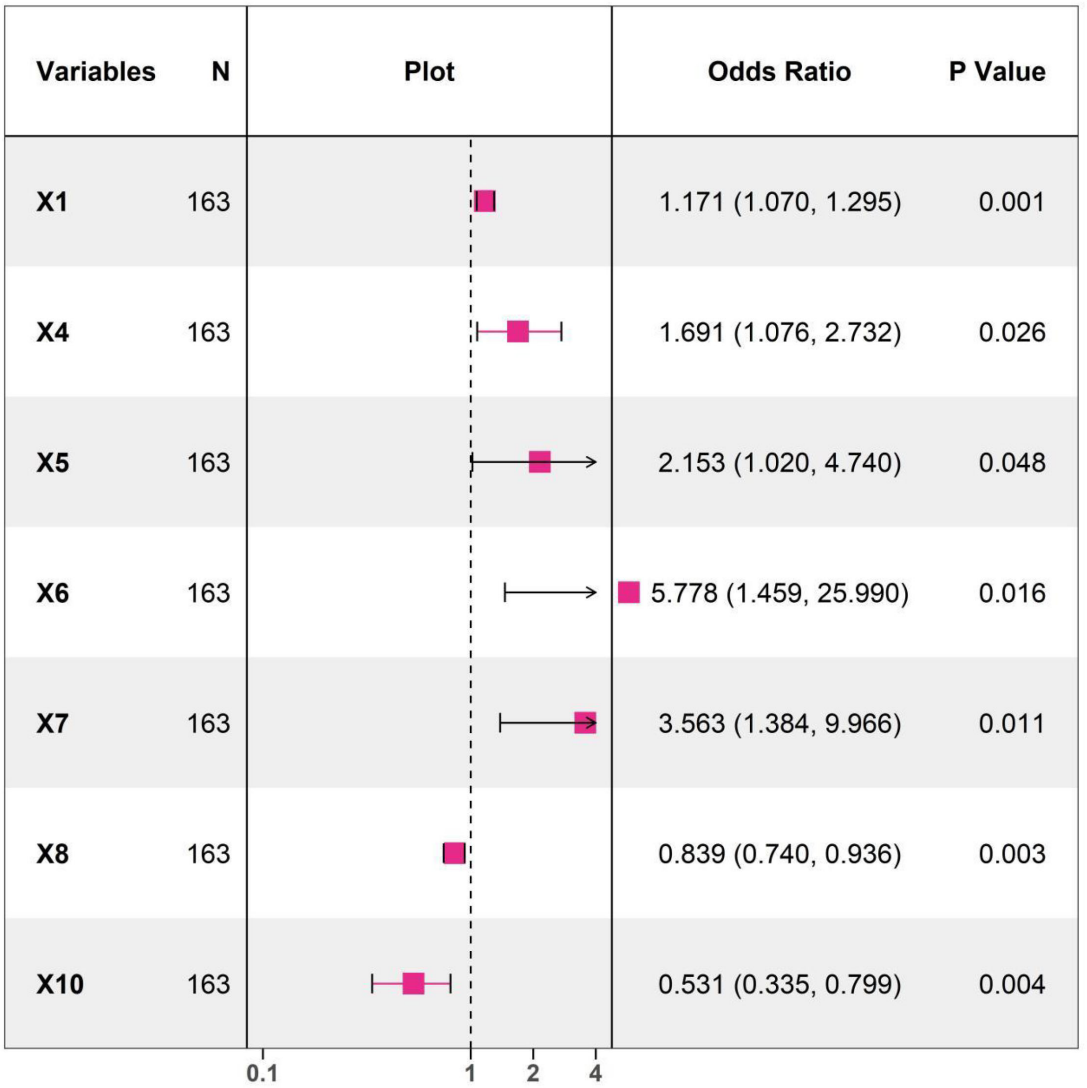
Forest plots of multivariate logistic regression analysis diagram (X1 Age, X4 Relative abundance of Fusobacterium nucleatum, X5 Relative abundance of Prevotella genus, X6 Relative abundance of Porphyromonas genus, X7 Relative abundance of Leptotrichia genus, X8 Relative abundance of Streptococcus genus, X10 Relative abundance of Actinobacteria genus).

Whether respondents experienced major cardiovascular endpoint events during the follow-up period was used as the dependent variable (non-event group=0, event group=1). The indicators with statistical significance in the univariate analysis were included in the LASSO regression for variable screening (**Supplementary Table 1**). Variables were selected using the screening criterion of lamda.1se (**Supplementary Figures 1**, **2**). The appropriate predictor variables were age, relative abundance of *Fusobacterium nucleatum, Prevotella*, *Porphyromonas*, *Leptotrichia*, *Streptococcus*, and *Actinomyces*. These indicators were included in the multivariate logistic regression analysis. The results showed that age, relative abundance of *Fusobacterium nucleatum, Prevotella, Porphyromonas*, and *Leptotrichia* were independent risk factors for respondents to experience major cardiovascular endpoint events, while relative abundance of *Streptococcus* and *Actinomyces* were independent protective factors for major cardiovascular endpoint events (all *P* < 0.05) (**Figure 2**; **Supplementary Table 2**).

### Prediction performance of machine-learning models in the training and validation set

**Figure 3:**
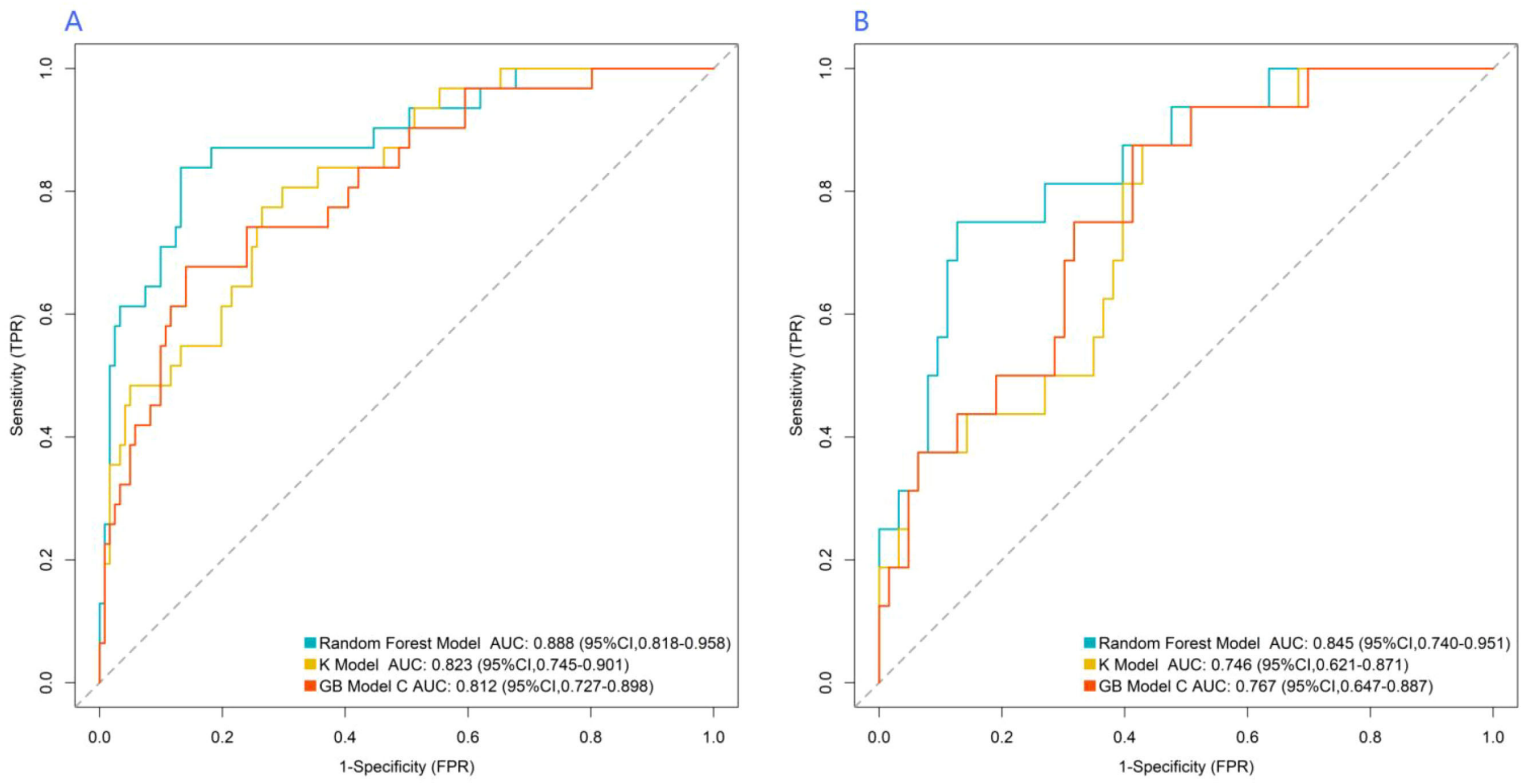
Receiver operating characteristic curve of machine-learning models [**(A)** training set, **(B)** validation set, AUC. Area under the curve; CI, confidence interval].

**Figure 4:**
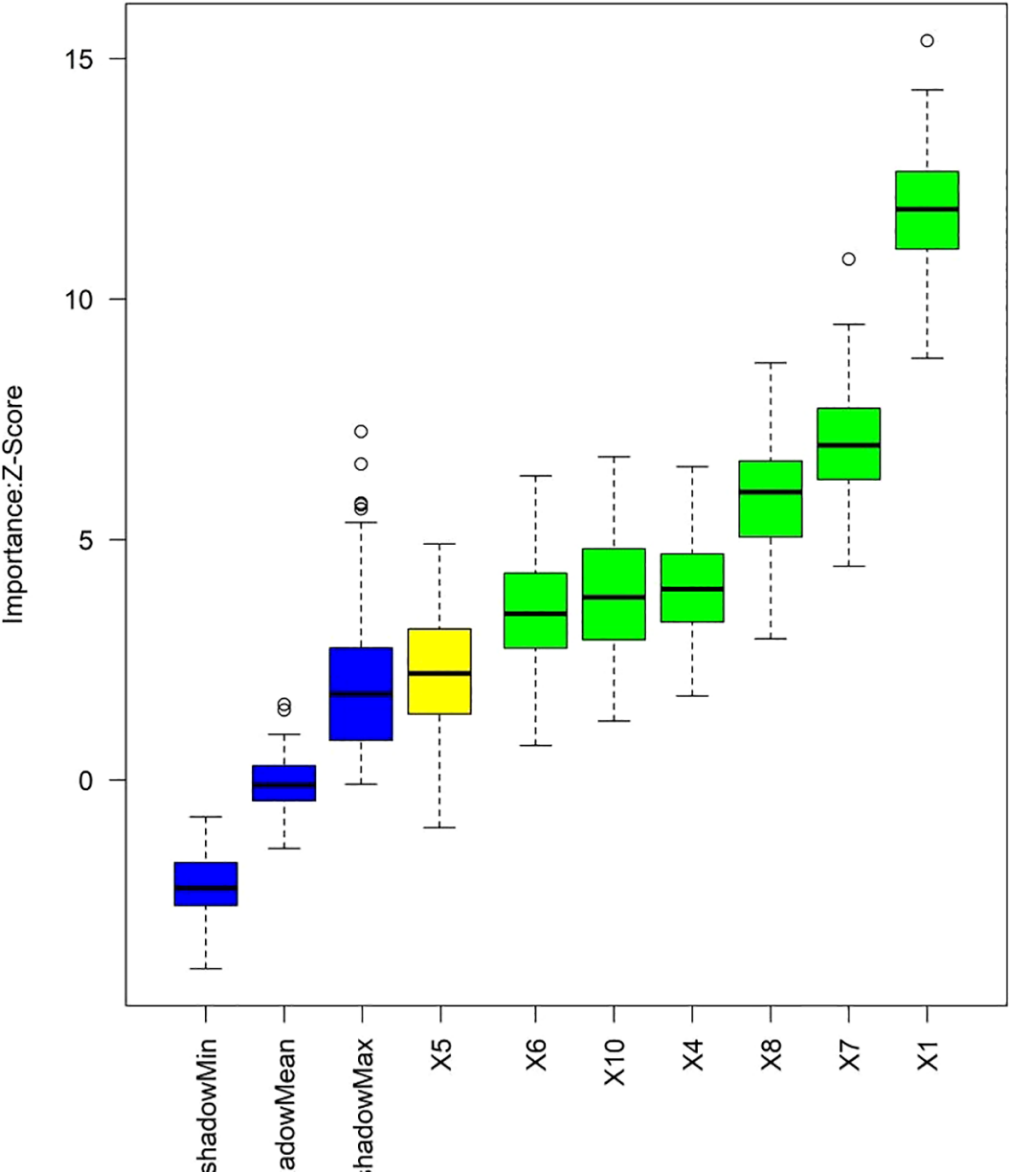
Z-score quantifies the contribution of each variable to the model prediction (X1 Age, X4 Relative abundance of Fusobacterium nucleatum, X5 Relative abundance of Prevotella genus, X6 Relative abundance of Porphyromonas genus, X7 Relative abundance of Leptotrichia genus, X8 Relative abundance of Streptococcus genus, X10 Relative abundance of Actinobacteria genus).

The RF, GB, and KNN algorithm model were used for prediction in the training and validation set. The AUC values of the three models in the training set were 0.888, 0.823, and 0.812 respectively, and the AUC values in the validation set were 0.845, 0.746, and 0.767 respectively. The model with the largest AUC value was selected as the optimal model for this study, which was the RF model (**Figure 3**). The Z-score was used to quantify the contribution of each variable to the model prediction (**Figure 4**). The results showed that variables such as age, relative abundance of *Leptotrichia* and *Streptococcus* (including the abundance of specific oral microbial taxa and clinical metabolic indicators) had significantly higher Z-scores, indicating that these variables had more prominent discriminative value for ASCVE prediction. In contrast, variables such as shadowMin and shadowMean had relatively low importance and limited contribution to the model prediction.

### Construction of a prediction model for major cardiovascular endpoint events in respondents

**Figure 5:**
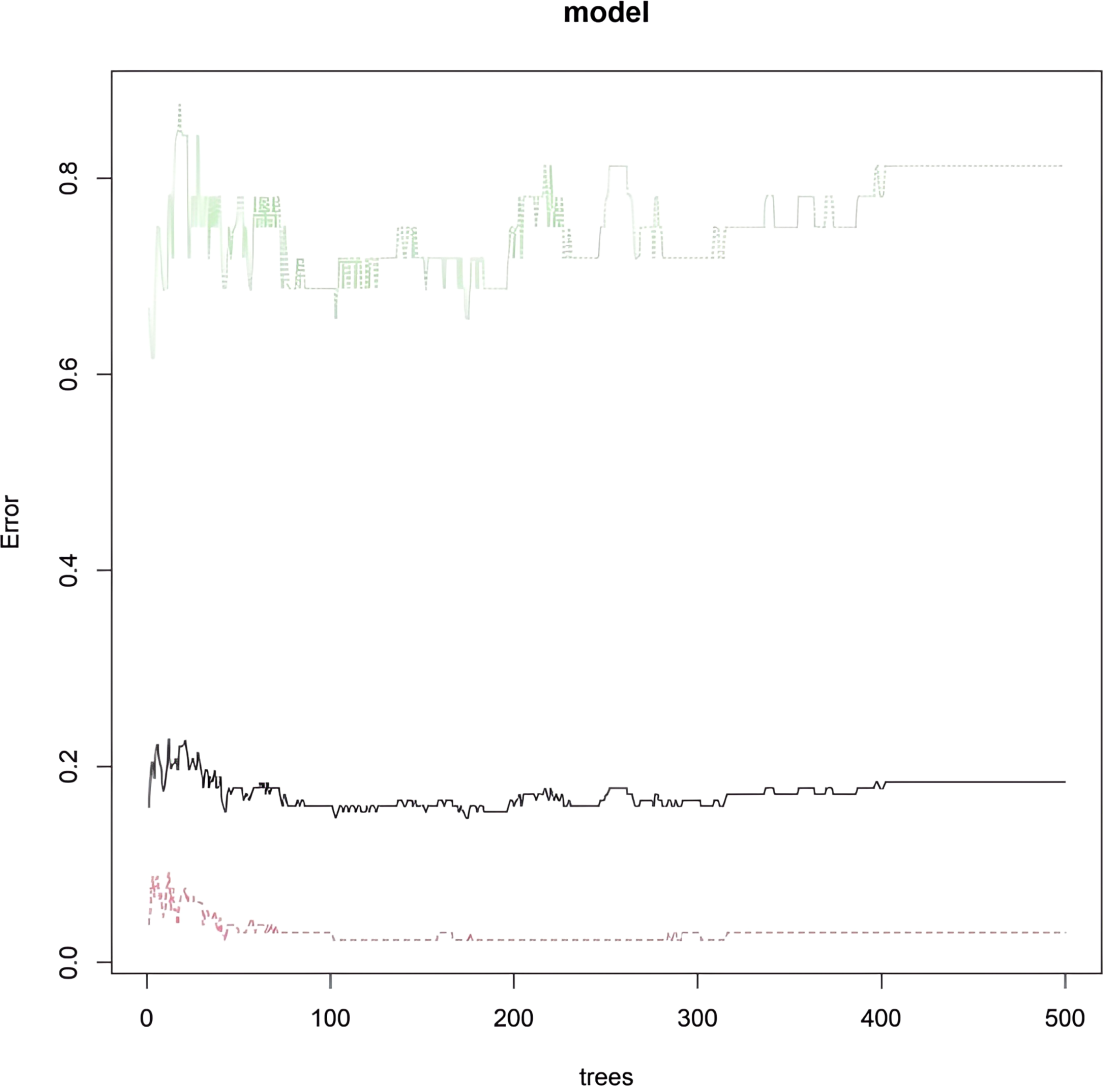
Trend of the average out-of-bag estimation error rate with the change in the number of decision trees.

**Figure 6:**
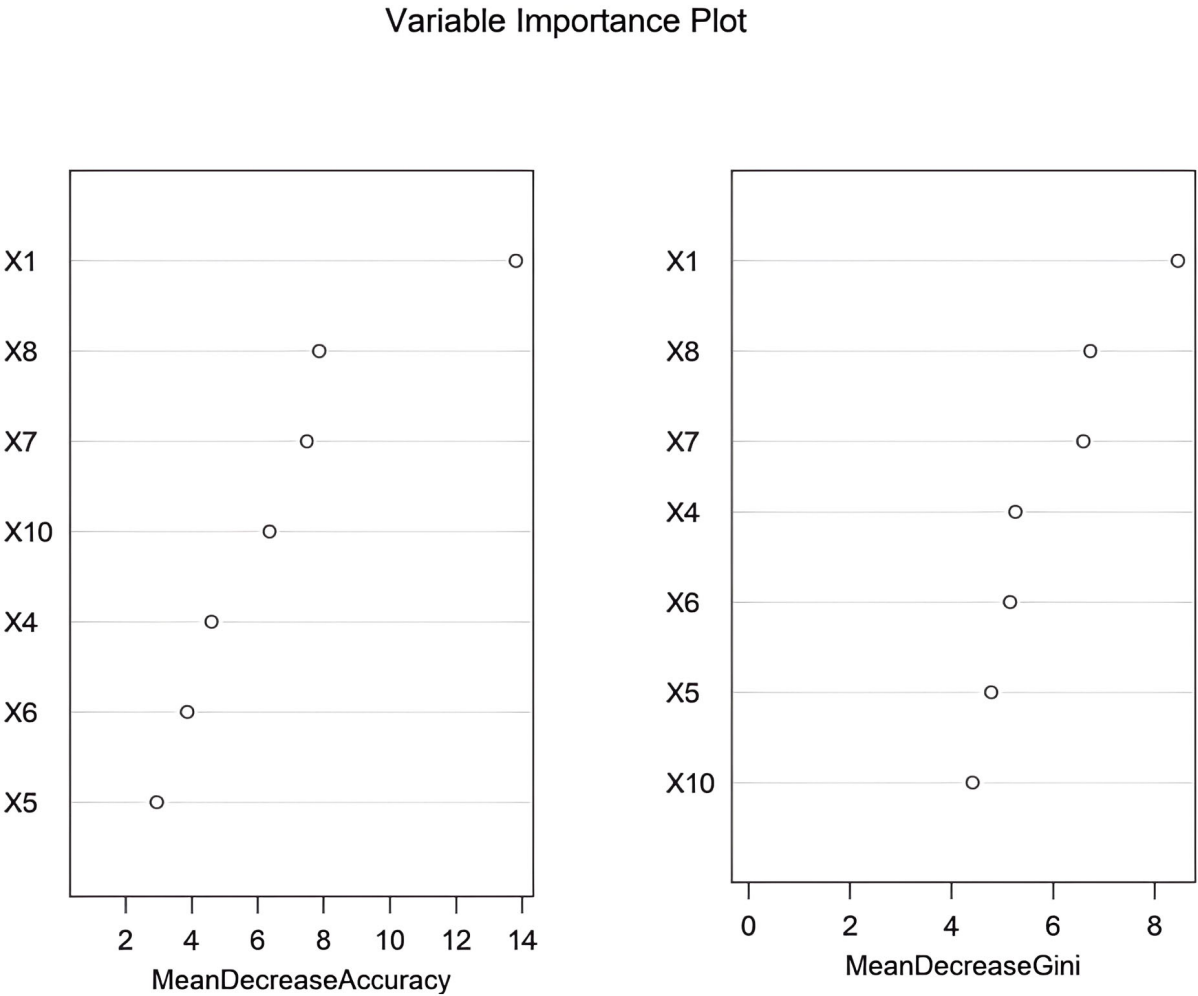
Importance ranking of the RF model (X1 Age, X4 Relative abundance of Fusobacterium nucleatum, X5 Relative abundance of Prevotella genus, X6 Relative abundance of Porphyromonas genus, X7 Relative abundance of Leptotrichia genus, X8 Relative abundance of Streptococcus genus, X10 Relative abundance of Actinobacteria genus).

**Figure 7:**
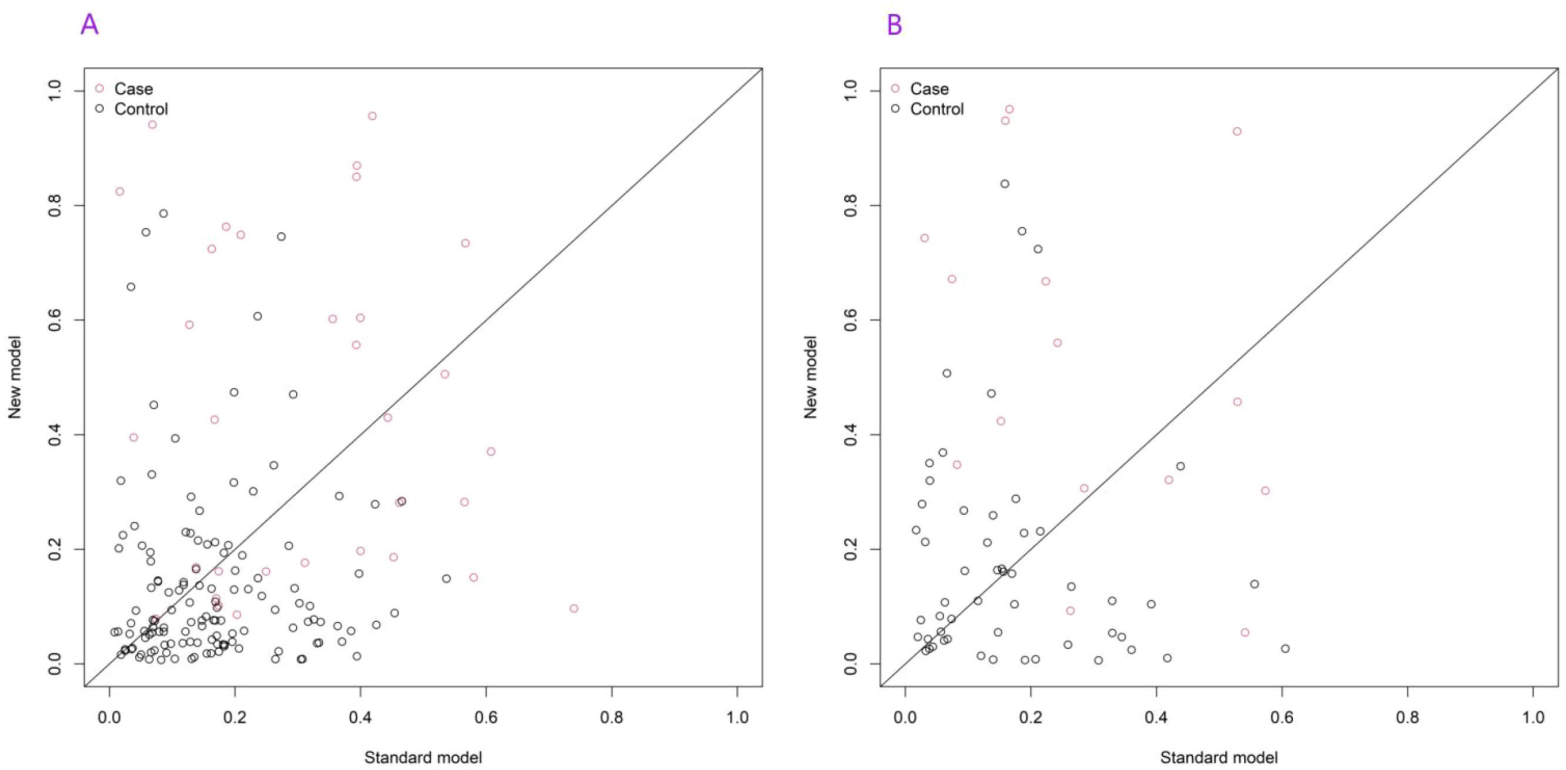
Continuous net reclassification improvement [**(B)** training set, **(B)** validation set].

As the number of decision trees increased, the overall error gradually stabilized. This change trend reflected the dynamic change characteristics of the prediction performance of the model during the iterative construction of decision trees. This trend could be used to assist in judging the convergence of the model. When the error curve tended to be stable, it indicated that the model complexity reached a certain level, and the optimization effect of adding new decision trees on the error was limited. This provided a parameter selection basis for determining the optimal number of decision trees and improving the model prediction efficiency, helping to select a configuration that could balance model complexity and prediction accuracy to enhance the model’s performance in predicting major cardiovascular endpoint events in respondents during the follow-up period (**Figure 5**). Based on the RF model, the importance scores of independent influencing factors for major cardiovascular endpoint events in respondents during the follow-up period were calculated. The importance ranking was as follows: age, relative abundance of *Streptococcus*, *Leptotrichia*, *Actinomyces*, *Fusobacterium nucleatum*, *Porphyromonas* and *Prevotella* (**Figure 6**). To comprehensively evaluate the re-classification ability of the integrated model, the continuous net re-classification index (Category-free NRI) was calculated. It did not depend on any risk threshold and only cared about the model for those who eventually experienced events. The continuous net re-classification index (NRI = 0.315, 95% CI: 0.233-0.862, *P* < 0.05) and the integrated discrimination improvement index (IDI = 0.227, 95% CI: 0.013-0.441, *P* < 0.05) were both statistically significant (**Figure 7**).

### Comparison with traditional clinical models

Traditional models based solely on traditional clinical risk factors (age, LDL-C, and current smoking status) were constructed. The same statistical methods as the integrated model were used for modeling, and its performance was verified in the same training and validation sets. For the traditional clinical model, the AUC values of the RF, GB and KNN algorithms in the training set were 0.721, 0.689, and 0.675, respectively; in the validation set, the AUC values of the three algorithms were 0.693, 0.658, and 0.642, respectively. Whether in the training set or the validation set, the AUC values of the traditional clinical model were significantly lower than those of the integrated model. Specifically, compared with the integrated RF model, the AUC of the traditional RF model decreased by 0.167 in the training set and 0.152 in the validation set (both *P* < 0.05). The continuous NRI of the traditional clinical model was 0.082 (95% CI: -0.031-0.195, *P* = 0.156), and the IDI was 0.053 (95% CI: -0.017-0.123, *P* = 0.138), neither of which was statistically significant, indicating that the traditional model had limited ability to reclassify ASCVD risk (**Table 3**).

**Table 3:** Summary of model performance comparison.

Model type	Algorithm	Training set AUC (95%CI)	Validation set AUC (95%CI)	NRI (95%CI)	IDI (95%CI)
Traditional clinical model	RF	0.721 (0.635-0.807)	0.693 (0.571-0.815)	0.082 (-0.031-0.195)	0.053 (-0.017-0.123)
	GB	0.689 (0.598-0.780)	0.658 (0.530-0.786)		
	KNN	0.675 (0.583-0.767)	0.642 (0.512-0.772)		
Integrated model	RF	0.888 (0.818-0.958)	0.845 (0.740-0.951)	0.315 (0.233-0.862)	0.227 (0.013-0.441)
	GB	0.823 (0.745-0.901)	0.746 (0.621-0.871)		
	KNN	0.812 (0.727-0.898)	0.767 (0.647-0.887)		

## Discussion

Through a retrospective cohort design, we confirmed that baseline oral microbiome composition (including relative abundance of specific genera) is significantly associated with future ASCVD risk. We screened out oral microbial markers with predictive value using machine learning algorithms. The integrated model combining oral microbial markers and traditional clinical factors showed better predictive performance and risk reclassification ability than the model based solely on clinical factors.

Our findings on the association between specific oral bacterial genera and ASCVD risk are consistent with existing research. For example, Nibali et al. reported that the abundance of Streptococcus and Actinomyces is correlated with metabolic syndrome (a key risk factor for ASCVD), and periodontal probing depth (reflecting oral inflammation) is positively correlated with left ventricular hypertrophy (a manifestation of ASCVD target organ damage) (Nibali et al., 2019; Nibali et al., 2023). This aligns with our result that oral microbiome dysbiosis is associated with ASCVD risk, further validating the “oral-systemic disease axis” (Barbour et al., 2022). In addition, existing studies have detected DNA of oral pathogenic bacteria such as Porphyromonas gingivalis and Fusobacterium nucleatum in atherosclerotic plaques, confirming that oral bacteria can participate in the development of atherosclerosis (Nearing et al., 2020; Linton et al., 2023)—this also supports our finding that Fusobacterium nucleatum and Porphyromonas are independent risk factors for MACE.

The multiple risk-related genera identified in this study (e.g., Fusobacterium nucleatum, Porphyromonas) are classic periodontal pathogens or oral opportunistic pathogens (Reyes-Soffer et al., 2022), and their potential mechanisms of promoting ASCVD may include two aspects: first, direct invasion—these bacteria can enter the bloodstream through oral ulcers or transient bacteremia caused by dental procedures, colonize damaged vascular endothelium, and directly participate in atherosclerotic plaque formation and destabilization (Sedghi et al., 2021); second, immune-inflammatory activation—persistent oral bacteria act as chronic antigenic stimuli, activating the systemic immune response and increasing levels of circulating inflammatory mediators (e.g., IL-1β, IL-6, TNF-α) and hs-CRP, thereby accelerating atherosclerosis (Embry and Rennicke, 2024; Nankivell et al., 2024). The increasing trend of hs-CRP in the event group (though not statistically significant) in this study supports this mechanism. For protective genera such as Streptococcus, their role may be related to maintaining oral microbiome balance, inhibiting pathogenic bacteria growth, and regulating host immune response (Al Samarraie et al., 2023; Porsch and Binder, 2024). From the perspective of clinical translation, the integrated model in this study has important application potential. The significant improvement in NRI and IDI indicates that the model can more accurately reclassify individuals into correct risk levels. For example, for those classified as “intermediate-risk” by traditional models, the integrated model may identify “truly high-risk” individuals by detecting their oral microbial characteristics, thereby recommending more active interventions (e.g., targeted oral hygiene management) while avoiding over-treatment of low-risk individuals (Nayor et al., 2021). This “microbiome score” based on key genera relative abundance shows potential as a non-invasive complementary risk assessment tool. However, its clinical application still requires further validation in larger cohorts, and combination with periodontal parameters (e.g., PPD) and GCF markers (e.g., IL-6) to improve predictive value (Nibali et al., 2022).

However, this study still has certain limitations. First, retrospective we only adjusted for major traditional risk factors (e.g., hypertension, dyslipidemia), unmeasured factors such as diet structure (e.g., differences between Chinese and Western diets, which may affect oral microbiome composition), oral hygiene habits, and socioeconomic status may simultaneously influence the oral microbiome and ASCVD risk (Nankivell et al., 2024). This limitation may restrict the extrapolation of our findings to global populations with diverse dietary patterns. Second, the sample size was relatively limited, especially the number of MACE events was only 40 cases, which is slightly lower than the estimated 50 cases in the sample size calculation. This may reduce the statistical power of the study and increase the risk of overfitting, thereby limiting the complexity, stability and generalizability of the model. Third, there are deficiencies in the description of microbiome data processing: specific parameters such as rarefaction depth and average reads per sample have not been clearly reported; relative abundance data were used directly without transformation (e.g., CLR), which may lead to biases in regression analysis due to the compositional nature of microbiome data. Fourth, although PCoA and beta-diversity were calculated in the methods, we did not report or discuss their results due to the difficulty in supplemental figure integration, which makes it impossible to fully clarify the differences in oral microbial community structure between the event group and non-event group. These issues need to be addressed by standardizing data processing and supplementing result reporting in future studies. Although LASSO regression and cross-validation were used to prevent overfitting, the final model still needs to be validated in a larger and more independent external cohort. Third, 16S rRNA sequencing was used. Although this method can effectively identify information at the genus level, it cannot provide accurate information at the species level or about functional genes (such as virulence factors) (Sopic et al., 2023). Fifth, hs-CRP is a non-specific systemic inflammatory marker that cannot capture the localized inflammatory response in the oral cavity, whereas GCF markers (e.g., IL-6, IL-8) are more specific to oral inflammation, we did not collect periodontitis-related clinical data or GCF samples, and the lack of these data may have underestimated the role of oral inflammation in ASCVD. In the future, metagenomic sequencing technology will be able to reveal its functional mechanisms more deeply.

Based on the above findings and limitations, Future research should focus on: (1) External validation: Validating the model in large independent cohorts from different regions and ethnic groups, especially those with diverse dietary habits (e.g., Western populations), to confirm its generalizability; (2) Mechanism exploration: Using animal models (e.g., ApoE-/- mice) to verify the causal role of high-risk genera (e.g., Fusobacterium) in atherosclerotic lesions; (3) Technology upgrade: Applying metagenomic sequencing to analyze microbial functional genes (e.g., virulence factors) and metabolomics to identify key metabolites; (4) Intervention research: Investigating whether improving oral microbiome (e.g., probiotics, professional oral care) reduces ASCVD risk, to provide new intervention targets (Nibali et al., 2022; Arnold and Koenig, 2025).

## Conclusion

This study confirmed that the oral microbiome is an independent predictor of ASCVD risk. Combining oral microbial markers with traditional clinical risk factors can significantly improve the early prediction ability of ASCVD events. The results of this study provide new ideas for the primary prevention strategy of cardiovascular diseases, that is, to achieve early risk identification and precise stratification through non-invasive oral microbiota detection, which has important public health significance and clinical translation prospects.

## Data Availability

The original contributions presented in the study are included in the article/**Supplementary Material**, further inquiries can be directed to the corresponding author/s.
