# Supplementary material for: An oral microbiome model for predicting atherosclerotic cardiovascular disease
Source: Front Cell Infect Microbiol. 2026 Jan 26;15:1707599. doi: 10.3389/fcimb.2025.1707599 (PMC12884645; doi:10.3389/fcimb.2025.1707599)
Supplement: Supplementary file 1 [file DataSheet1.docx]

**Supplemental Table 1 .** Variable assignment table.

| Variable | Meaning | Assignment |
| --- | --- | --- |
| X1 | Age | Continuous variable |
| X2 | Low-density lipoprotein cholesterol | Continuous variable |
| X3 | Current smokers | 0 = No, 1 = Yes |
| X4 | Relative abundance of Fusobacterium nucleatum | Continuous variable |
| X5 | Relative abundance of Prevotella | Continuous variable |
| X6 | Relative abundance of Porphyromonas | Continuous variable |
| X7 | Relative abundance of Leptotrichia | Continuous variable |
| X8 | Relative abundance of Streptococcus | Continuous variable |
| X9 | Relative abundance of Rothia | Continuous variable |
| X10 | Relative abundance of Actinomyces | Continuous variable |
| Y | Whether major cardiovascular endpoint events occurred | 0 = Non-event group, 1 = Event group |


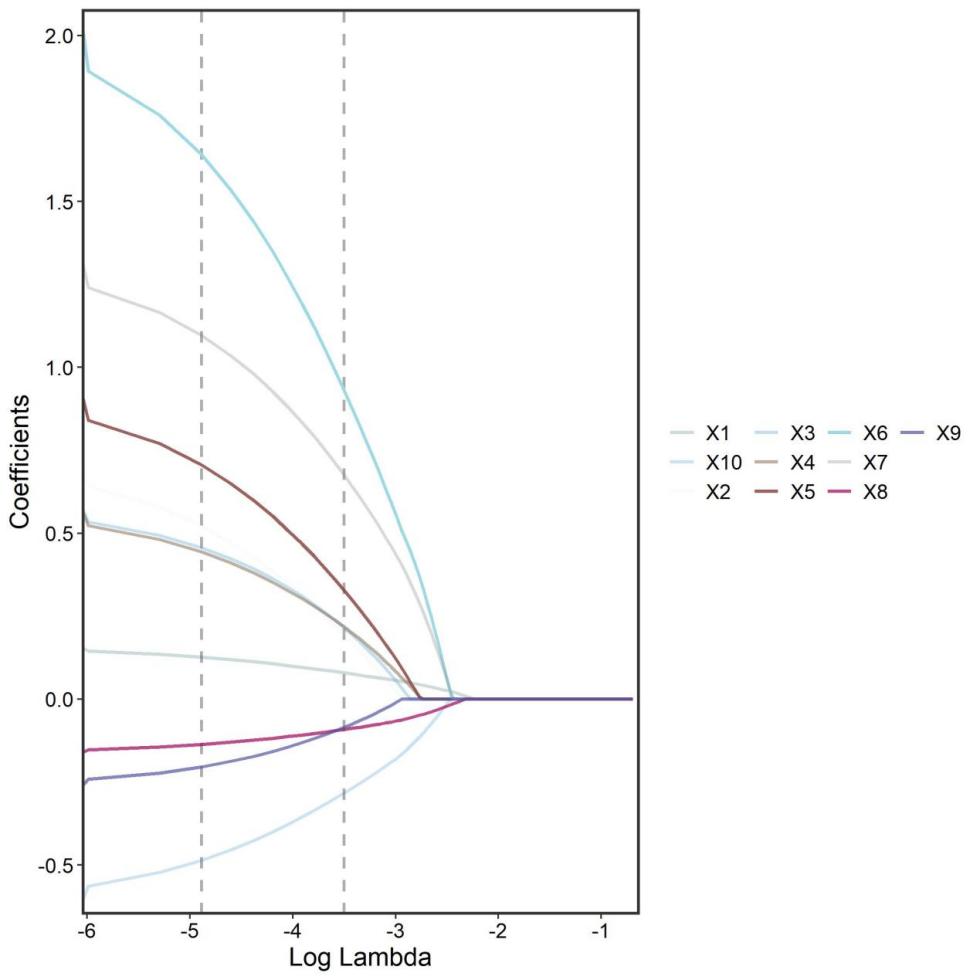


**Supplemental Figure 1.** LASSO regression analysis diagram.

**Supplemental Table 2.** Detailed results of multivariate Logistic regression analysis.

| Indicators | B | Standard error | *P* | OR | 95% Confidence Interval |
| --- | --- | --- | --- | --- | --- |
| Age | 0.158 | 0.049 | 0.001 | 1.171 | 1.070-1.295 |
| Relative abundance of Fusobacterium nucleatum | 0.526 | 0.231 | 0.026 | 1.691 | 1.076-2.732 |
| Relative abundance of Prevotella | 0.768 | 0.389 | 0.048 | 2.153 | 1.020-4.740 |
| Relative abundance of Porphyromonas | 1.755 | 0.732 | 0.016 | 5.778 | 1.459-25.990 |
| Relative abundance of Leptotrichia | 1.269 | 0.453 | 0.011 | 3.563 | 1.384-9.966 |
| Relative abundance of Streptococcus | -0.177 | 0.062 | 0.003 | 0.839 | 0.740-0.936 |
| Relative abundance of Actinomyces | -0.633 | 0.231 | 0.004 | 0.531 | 0.335-0.799 |


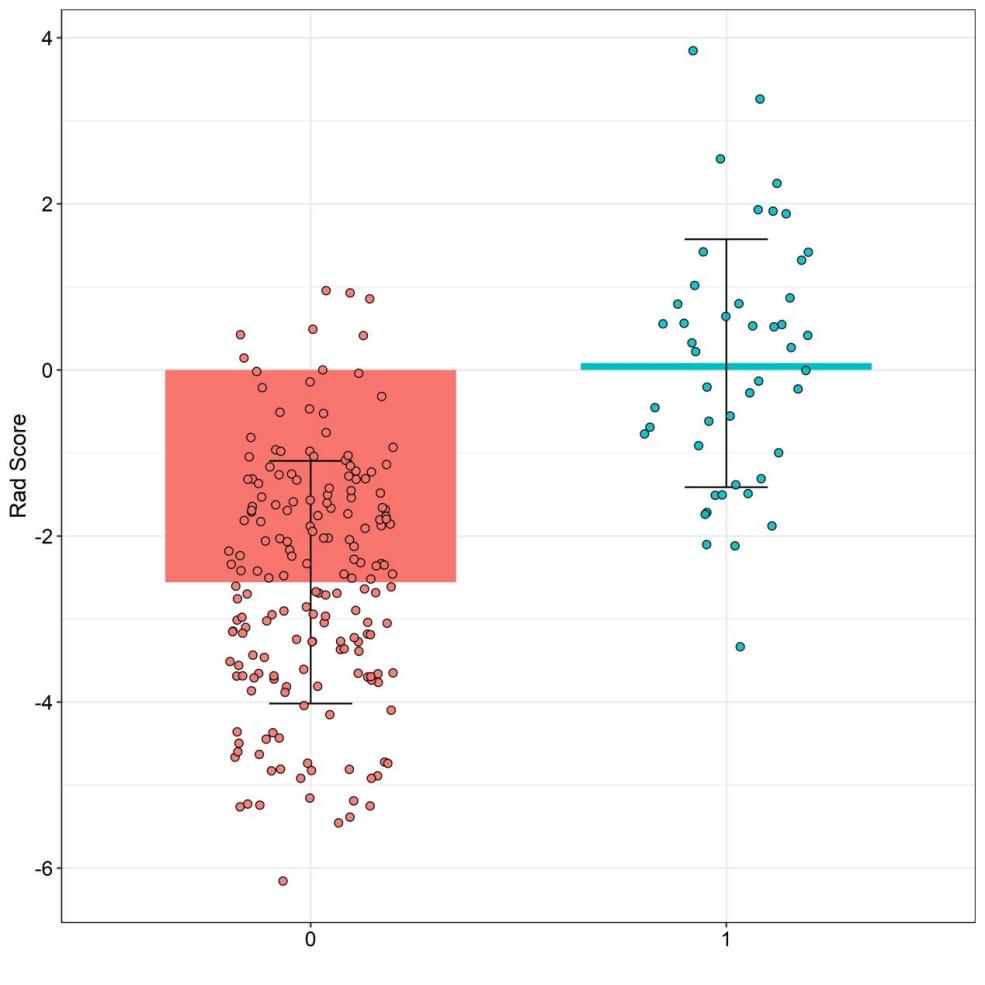


**Supplemental Figure 2.** Comparison of LASSO score differences.
